# Multicentric prospective study of effect of dietary intake on quality of life for patients with end-stage cancers

**DOI:** 10.20407/fmj.2018-023

**Published:** 2019-11-02

**Authors:** Miyo Murai, Takashi Higashiguchi, Hiroshi Ohara, Nagato Katsura, Akihiko Futamura, Naomi Nakayama

**Affiliations:** 1 Department of Surgery and Palliative Medicine, Fujita Health University, School of Medicine, Toyoake, Aichi, Japan; 2 The University of Shimane, School of Nursing and Nutrition, Matsue, Shimane, Japan

**Keywords:** Dietary energy intake, Quality of life, Terminal cancer patients, Palliative care

## Abstract

**Objective:**

Impaired dietary intake (DI) contributes to deterioration of quality of life (QOL) in patients with end-stage diseases, including cancer, but the effects of DI on QOL specifically in terminal cancer has not been widely studied. Here, we evaluated the relationship between DI and QOL in patients with end-stage cancers.

**Methods:**

We evaluated the energy amount of DI, performance status (PS) and QLQ-C15-PAL score of cancer patients with short prognoses in multicentre survey and analysed the parameters that influence QOL.

**Results:**

We recruited 33 patients in this study. In univariate analysis, DI was significantly associated with PS (*P*=0.002, *r*=–0.531), physical functioning (*P*=0.003, *r*=–0.503), fatigue (*P*=0.038, *r*=–0.362), and appetite loss (*P*=0.004, *r*=–0.490).

**Conclusions:**

Improved DI could contribute to QOL of patients with end-stage cancers.

## Introduction

Patients with advanced cancers often develop impaired dietary intake (DI) for various reasons, including loss of appetite, oral problems, gastrointestinal tract obstruction and swallowing disorders associated with decreased skeletal muscle mass.^1–4^ DI and nutritional status are thought to greatly influence the quality of life (QOL) and the efficacy of anti-cancer therapies.^5^ Therefore, maintaining DI and good nutritional status is crucial for these patients.^5,6^ However, patients with end-stage cancers often become cachexic, and may develop a refractory, late-stage cachexia for which nutritional therapy is no longer effective.^7–9^ Nevertheless, even for these patients, nutritional support is apparently important in maintaining QOL. Unfortunately, few studies have evaluated the impact of DI on QOL in end-stage cancer. This kind of study is difficult for two reasons. One reason is that assessing health-related QOL is especially challenging for terminally ill patients. The QLQ-C30 questionnaire, which was created by the European Organisation for Research and Treatment of Cancer (EORTC), is widely utilized for health-related QOL evaluation worldwide,^4,10,11^ but can be burdensome for patients with end-stage cancer to complete. In this situation, the QLQ-C15-PAL,^12,13^ which is a shorter version of original QLQ-C30, has been used for QOL evaluation in palliative care settings.^14,15^ The other reason is that measuring patients’ whole nutritional intake is quite difficult, as it varies and is often disturbed, especially during terminal stages.

In this study, we used the QLQ-C15 for QOL evaluation, and assessed nutritious intake properly with the cooperation of patients and medical staffs. We then examined how DI was related to QOL in patients with end-stage cancers.

## Methods

### Patients

This study included patients with end-stage cancers who were admitted to Japanese medical facilities dedicated to palliative care and nutritional support between October 2012 and February 2013. All patients met the following selection criteria: (a) physician-predicted life expectancy of approximately 1 month; (b) able to ingest food at the start of the survey; and (c) capable of understanding the informed consent document and the QOL survey description. Patients whose causes of death were unrelated to cancer progression or who were alive at the end of the study were excluded.

### Study design

A physician, registered dietitian or nurse performed the QLQ-C15-PAL questions, surveys for nutritional intake evaluation, and Eastern Cooperative Oncology Group performance status (PS) once a week from the start of the study.

### Nutritional intake

Patients’ nutritional intake consisted of what was provided orally, enterally, and parenterally. Amounts of enteral and parenteral nutrition were calculated based on medical records. Total oral intake was measured as the sum of provided hospital food, food brought from outside of the hospital and dietary supplements.

### EORTC QLQ-C15-PAL

Patients’ QOL was evaluated using the QLQ-C15-PAL questionnaire, with prior permission of the EORTC. The QLQ-C15-PAL consists of two functional scales that included physical (questions 1–3) and emotional (questions 13 and 14) functioning, and symptom scales that included pain (questions 5 and 12) and fatigue (questions 7 and 11). It also includes several single questions about dyspnea (4), insomnia (6), appetite loss (8), nausea (9) and constipation (10) and one global QOL item (question 15). For questions 1–14, patients responded to a four-point Likert scale: 1) not at all, 2) a little, 3) quite a bit and 4) very much. For question 15 (global QOL), patients respond to a seven-point numerical scale from very poor to excellent overall QOL. Questionnaire data were processed according to the procedures outlined in the EORTC QLQ-C30 scoring manual and the addendum for scoring the QLQ-C15-PAL.^13^

### Performance status

PS was assessed according to the Eastern Cooperative Oncology Group scale.

### Ethical approval

This study was conducted as part of the 2012 Geriatric Health promotion project, “A study on the nutritional management for terminal ill cancer patients,” in compliance with the ethical principles of the Declaration of Helsinki and the “Ethical Guidelines for Epidemiological Studies” (Ministry of Education, Culture, Sports, Science and Technology and Ministry of Health, Labour and Welfare), and was conducted under the auspices of the project. The research protocol was evaluated and approved by the ethics committees of the Fujita Health University School of Medicine (registry no. 12-133) and of each facility.

### Statistical analysis

In this study we analysed the data at the start of the survey. All data were analysed using GraphPad PRISM 6 (GraphPad Software, San Diego, California, U.S.A.) or the Software Package for Social Sciences (SPSS) for Windows version 21.0 (IBM Company, Chicago, Illinois, USA). Data were evaluated for normality of distribution using the Shapiro–Wilk test. The descriptive statistics of normal distribution are reported as the mean±standard deviation, and non-normal distribution data are expressed as medians and interquartile range (IQR, i.e., between the 75th and 25th percentiles). Correlation assessment between single items was obtained using the Spearman correlation coefficient *r*, and was adopted for interpreting absolute values of correlation coefficients: *r*<0.1–0.3, weak; 0.3–0.5, moderate and >0.5, strong.

## Results

### Completion of questionnaires

The survey was administered to 50 patients once a week in 15 participating medical facilities. Five patients who lack the full assessment and 12 who were alive at the end of the study period were excluded. Accordingly, 33 patients were selected.

### Patient characteristics

Data for 33 patients were available. Table 1 shows patient characteristics at the start of the survey. The study population comprised 13 women and 20 men, with a median age of 71.0 years. Their primary cancer sites were the colorectum (*n*=8, 24.2%), stomach (*n*=4, 12.1%), lung (*n*=4, 12.1%) and liver (*n*=4, 12.1%). Their PS status were PS3 (*n*=13, 39.4%), PS2 (*n*=8, 24.2%), PS4 (*n*=8, 24.2%) and PS1 (4, 12.1%). Median survival was 21 days, and the median number of evaluations per patient was 4 (range: 1–7). The dietary energy intake ranged from 150 to 870 kcal/day, with a median of 500 kcal/day at the start of the survey. Twenty of the 33 patients received supplementary intravenous transfusion; their median hydration volume and nutritional energy were 500 mL/day and 205 kcal/day, respectively. Five patients received supplemental enteral nutrition via nasogastric tube or percutaneous endoscopic gastrostomy; their median volume of enteral nutrition was 137 ml/day (250 kcal/day). The median total energy intake of dietary, intravenous and enteral nutrition was 680 kcal/day.

### DI and PS, EORTC QLQ-C15-PAL score, prognosis

**Figure 1:**
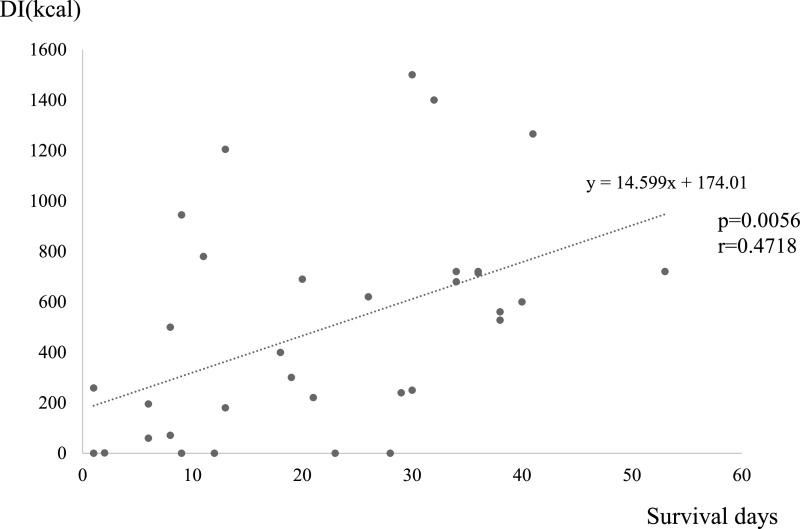
Correlation between DI and survival days.

Results for the patients’ EORTC QLQ-C15-PAL questionnaires are shown in Table 2. Correlations between DI and PS, the EORTC QLQ-C15-PAL score are presented in Table 3. In univariate analysis DI was significant associated with PS, physical functioning, fatigue and appetite loss. However, in multivariate analysis, DI was significantly associated with survival days (*P*=0.0056, *r*=0.4718; Figure 1).

## Discussion

To our knowledge, this is the first study to quantitatively evaluated the relationship between DI and QOL in patients with end-stage cancers. Whereas DI has been shown to greatly influence QOL of patients with end-stage cancer,^10,11,16–18^ we have found that DI is positively related to PS, physical functioning, fatigue and appetite loss.

Although evaluation of QOL is usually performed with a questionnaire that assesses multiple parameters, the standard questionnaire is often burdensome for patients with terminal diseases.^19,20^ The EORTC QLQ-C15-PAL is a shortened version of the EORTC QLQ-C30, and was recently developed specifically for patients who received palliative care.^12,13^ The reliability of EORTC QLQ-C15-PAL for the assessment of QOL has been validated in some research papers,^21–23^ including one from Japan.^24^

Due to their disturbed appetites, patients with end-stage cancer tend to eat only small amounts of foods that meet their individual preferences, which may be provided by the hospital, family or friends. In addition, some patients receive intravenous nutrition or enteral feeding. Therefore, accurate evaluation of nutritional intake is often difficult for patients with end-stage cancer, which makes this sort of study challenging.^25,26^ In this study, we successfully obtained detailed information of nutritional intake from individuals through inquiring surveys and the precise medical records of each hospital. One reason behind this attainment is that nutrition support teams operate efficiently in most Japanese medical facilities.^27^ They practice precise nutritional screening and offer nutritional support for patients, including those who are terminally ill.

This study quantitatively showed that DI of patients with end-stage cancer was associated with several factors related to QOL. DI was positively correlated with physical functioning scores, and inversely correlated with several distressing symptoms such as fatigue and appetite loss.

The impact of nutritional support on cancer patients who receive anti-cancer therapy has been widely studied and is considered effective, as patients with good nutritional condition have better therapeutic responses and less side effects.^5,6,28–31^However, few studies have evaluated the influence of nutritional support on cancer patients under palliative care.^32,33^ Lee et al. reported that improved oral feeding independence in end-stage cancer patients led to better activities of daily living and QOL,^33^ Our finding reinforces their result of the importance of maintaining DI even at the end of life. Yavuzsen et al. reported that gastrointestinal symptoms such as early satiety, taste changes, food aversions, and altered sense of smell were important factors in anorexia and impaired food intake.^34^ However, we found no correlation between DI and gastrointestinal symptoms, including nausea, vomiting and constipation, but found a negative correlation between DI and appetite loss. This might be attributed to our inclusion only of patients who were still capable of ingesting food at the start of the survey and exclusion of patients at a risk of gastrointestinal obstruction.

QOL of cancer patients is affected by their PS.^18,35^ As death from cancer progression approaches, QOL and PS generally deteriorate, and DI declines.^26^ Dieher et al. reported that for patients with end-stage cancer, QOL was better than expected until the final 3 weeks of life, when a terminal drop of QOL was observed. They also mentioned that the baseline health status was not related to the length of good QOL. In this situation, intervention to improve QOL should be started at least before the last 3 weeks of life. According to our finding, DI is closely associated with PS and physical functioning. Maintaining DI would help delay their deterioration. To secure DI as much as possible, proper intervention and symptom control are required to alleviate impaired appetite. Installation of digestive tract stents and medication for controlling nausea and constipation should be considered.^36^

When patients suffer intra-oral problem, gastrointestinal stenosis or dysphagia, adjustment of food texture is necessary. In this context, appetizing newly developed soft foods are worth mentioned. These foods have the same appearance as ordinary meals. but are scientifically processed to be swallowed without mastication and easily digested.^37^ This is particularly notable as a useful alternative for maintaining DI in patients with end-stage cancer.

Some reports are converse to our findings in terms of DI and survival. Hutton et al. reported that DI in advanced cancer patients did not improve their life expectancy.^38^ Unlike their finding, our study showed that DI was moderately associated with survival. This may be because we only enrolled patients with short expected survival at the start of this survey, whereas Hutton et al. recruited patients with advanced cancers, regardless of expected survival. Median survival of their patients and our patients were 7.8 months and 21 days respectively. This might be the reason for the differing results.

When we treat patients with end-stage cancer, cachexia should be considered. Cancer cachexia is a multifactorial syndrome characterized by skeletal muscle loss leading to progressive functional impairment and severely decreased QOL.^7,39^ Cachexia affects 50–80% of cancer patients in this setting,^40^ and dramatically affects QOL. Precise early identification followed by proper polymodal intervention, including optimal cancer therapy, symptom management, nutrition support, exercise, and psychological support are thought to be essential for cachexia patients.^40^

Enteral and parenteral loading are considered to be artificial nutritional support, whereas oral diet is considered a natural way of consuming nutrition. Refractory cachexia is characterized by its late stage and poor response to nutritional support and decreased survival. For patients with refractory cachexia, the benefits of nutritional therapy are limited. Excessive enteral and parenteral nutritional administration can even have harmful consequences such as oedema and fatigue. For these reasons, we often need to refrain from artificial nutritional loading for patients with refractory cachexia. In contrast, oral diet is usually taken at patients’ desire on a voluntary basis and rarely causes negative effect on patients’ condition, even for patients with refractory cachexia. Therefore, when patients develop refractory cachexia, the aim of nutritional support should be switched from improvement of nutritional status to maintenance of QOL. This is called a “gear-change”; artificial nutritional loading should be withdrawn at about this point.^41^

When estimated life expectancy is shorter than 3 months, patients are likely to be in the refractory stage of cachexia,^7^ This applies to most patients in this study because median survival was 21 days with an IRQ of 9 to 34 days. Intriguingly, DI was associated with better QOL, survival and PS in this study, with moderate significance.

This study has several limitations. First, we did not enrol all consecutive cancer patients at each facility during the study period. Because participating in experimental studies can be stressful for these patients, asking for their reluctant participation may be inappropriate. Second, as this study was conducted prospectively, the number of surveys for each patient was non-uniform due to the variations in observation periods. Third, because the number of enrolled patients was small, we were unable to fully evaluate the effects of intravenous or enteral nutrition. Our findings should be considered preliminary until validated in future studies.

In conclusion, this study showed evidence that DI affects QOL of cancer patients at the end of life. PS and survival are also positively correlated with DI and have interactive effects on QOL. Thus, DI helps improve QOL of patients with end-stage cancer. We believe that our study sheds light on DI and will increase awareness of its importance for these patients.

## Figures and Tables

**Table1:** Patients’ characteristics

Characteristics	
Age, years, (mean±SD)	68.5±10.6
Sex, n (%)	
Male	20
Female	13
Body weight, kg, (mean±SD)	52.3±12.1
Cancer primary site, n (%)	
Colorectal	8 (24.2)
Stomach	4 (12.1)
Lung	4 (12.1)
Liver	4 (12.1)
Pancreas	3 (9.1)
Head and neck	2 (6.1)
Biliary	2 (6.1)
Others	6 (18.2)
Metastasis, n (%)	
Liver	16 (48.5)
Lung	10 (30.3)
Bone/spine	9 (27.3)
Peritoneum	9 (27.3)
Brain	3 (9.1)
Others	7 (21.2)
Performance status, n (%)	
1	4 (12.1)
2	8 (24.2)
3	13 (39.4)
4	8 (24.2)
Survival, days, median (IQR)	21 (9–34)
Dietary energy intake, kcal/day, median (IQR)	500 (126–720)
Hydration volume via PN, ml/day, median (IQR) n=20	500 (500–1000)
Energy intake via PN, kcal/day, median (IQR) n=20	205 (69–420)
Hydration volume via EN, median (IQR) n=5	137 (20–258)
Energy intake via EN, kcal/day, median (IQR) n=5	250 (112–544)
Total energy intake, kcal/day, median (IQR)	680 (211–1050)

PN: parenteral nutrition, EN: enteral nutrition, IQR: interquartile range.

**Table2:** EORTC QLQ-C15-PAL scale (n=33)

EORTC QLQ-C15-PAL scale	Median
Physical functioning	10 (3–12)
Emotional functioning	3 (2–8)
Fatigue	6 (2–8)
Nausea and vomiting	1 (1–3)
Pain	4 (2–8)
Dyspnea	2 (1–4)
Insomnia	2 (1–4)
Appetite loss	3 (1–4)
Constipation	1 (1–4)
Global quality of life	4 (2–6)

**Table3:** Univariate and multivariate analysis with correlations between DI

	univariate			multivariate	
	p	r		p	r
PS	0.0015	–0.5312		0.1258	–0.3326
Physical functioning	0.0029	–0.5026		0.3662	–0.2151
Emotional functioning	0.1463	–0.2585			
Fatigue	0.0384	–0.3621		0.3569	0.2121
Nausea and vomiting	0.29	–0.1898			
Pain	0.38	–0.1579			
Dyspnea	0.9168	0.0189			
Insomnia	0.1244	–0.2729			
Appetite loss	0.0038	–0.4903		0.0588	–0.3863
Constipation	0.6973	–0.0703			
global QOL	0.4204	0.1451			
